# Genomic prediction modeling of soybean biomass using UAV‐based remote sensing and longitudinal model parameters

**DOI:** 10.1002/tpg2.20157

**Published:** 2021-09-30

**Authors:** Yusuke Toda, Akito Kaga, Hiromi Kajiya‐Kanegae, Tomohiro Hattori, Shuhei Yamaoka, Masanori Okamoto, Hisashi Tsujimoto, Hiroyoshi Iwata

**Affiliations:** ^1^ Graduate School of Agricultural and Life Sciences The Univ. of Tokyo 1‐1‐1 Yayoi Bunkyo Tokyo 113–8657 Japan; ^2^ Institute of Crop Science National Agriculture and Food Research Organization 2‐1‐2 Kannondai Tsukuba Ibaraki 305–8518 Japan; ^3^ Research Center for Agricultural Information Technology National Agriculture and Food Research Organization Kintetsu Kasumigaseki Building, 3‐5‐1 Kasumigaseki Chiyoda Tokyo 100‐0013 Japan; ^4^ Center for Bioscience Research and Education Utsunomiya Univ. 350 Minecho Utsunomiya Tochigi 321–8505 Japan; ^5^ Arid Land Research Center Tottori Univ. 1390 Hamasaka Tottori 680‐0001 Japan

## Abstract

The application of remote sensing in plant breeding can provide rich information about the growth processes of plants, which leads to better understanding concerning crop yield. It has been shown that traits measured by remote sensing were also beneficial for genomic prediction (GP) because the inclusion of remote sensing data in multitrait models improved prediction accuracies of target traits. However, the present multitrait GP model cannot incorporate high‐dimensional remote sensing data due to the difficulty in the estimation of a covariance matrix among the traits, which leads to failure in improving its prediction accuracy. In this study, we focused on growth models to express growth patterns using remote sensing data with a few parameters and investigated whether a multitrait GP model using these parameters could derive better prediction accuracy of soybean [*Glycine max* (L.) Merr.] biomass. A total of 198 genotypes of soybean germplasm were cultivated in experimental fields, and longitudinal changes of their canopy height and area were measured continuously via remote sensing with an unmanned aerial vehicle. Growth parameters were estimated by applying simple growth models and incorporated into the GP of biomass. By evaluating heritability and correlation, we showed that the estimated growth parameters appropriately represented the observed growth curves. Also, the use of these growth parameters in the multitrait GP model contributed to successful biomass prediction. We conclude that the growth models could describe the genetic variation of soybean growth curves based on several growth parameters. These dimension‐reduction growth models will be indispensable for extracting useful information from remote sensing data and using this data in GP and plant breeding.

## Core Ideas

- Application of growth models and unmanned aerial vehicle (UAV) remote sensing in genomic prediction was investigated.
- The growth of soybean plants was estimated from canopy area and height using a UAV.
- Growth models were used to determine growth patterns of the breeding population.
- The inclusion of growth parameters improved prediction accuracy of biomass.

**Abbreviations**
CA: canopy area
CH: canopy height
DSM: digital surface model
GP: genomic prediction
GSP: growth start point
GTP: growth termination point
HU: heat unit
NIR: near‐infrared
PH: plant height
RMSE: root mean squared errors
RRMSE: ratio of RMSE to mean value
UAV: unmanned aerial vehicle
WS: water‐stressed
WW: well‐watered

## 1 INTRODUCTION

The phenotype of a plant at harvest is a result of its genotype and environment. From a plant physiology or crop growth modeling viewpoint, plant phenotype at harvest can be expressed as a result of the accumulation and translocations of carbon production throughout their growth (Soltani & Sinclair, 2012). Therefore, it is expected that the estimation or prediction of plant traits at harvest will improve in accuracy if the growth processes are appropriately accounted for in the models. This hypothesis can also be applied to genomic prediction (GP) models, which predict genotypic values (breeding values) from genome‐wide marker data using statistical methods (Meuwissen et al., 2001). Recently, some extensions of GP models were proposed that incorporated plant growth processes (Technow et al., 2015; Toda et al., 2020). These reports suggested that the inclusion of growth processes into the GP models may improve the prediction accuracy and further the applications of GP in plant breeding (Technow et al., 2015).

To model growth processes in GP appropriately, sufficient data about the growth patterns of the target plant population are essential. However, there is a high labor cost and limited time to measure plants during growth in a breeding program, if relying on hand measurements and visual judgments. Recent technological developments have yielded an alternative phenotyping procedure (i.e., remote sensing). For plant breeding, remote sensing with ground vehicles (White et al., 2012) or aerial platforms such as unmanned aerial vehicles (UAVs) (Yang et al., 2017) were used to evaluate genetic variation in plant growth. From this work, it has been shown that remote sensing could capture the variation in a breeding population (Watanabe et al., 2017). Also, GP models that use a time‐series of remote sensing data could improve the prediction accuracies of wheat (*Triticum aestivum* L.) yields (Rutkoski et al., 2016). Therefore, the accuracy of GP is expected to improve by including longitudinal growth processes of plants as measured by remote sensing.

Several approaches can be applied to include remote sensing data into GP models. A multitrait GP model (Calus & Veerkamp, 2011; Jia & Jannink, 2012) has been recently used to incorporate several traits into the GP model (Crain et al., 2018; Rutkoski et al., 2016). By leveraging correlation among traits, a multitrait GP model could improve the prediction accuracies of those traits with low heritability (Jia & Jannink, 2012). However, the accurate estimation of the genetic covariance matrix among the traits may be difficult if the number of traits being measured becomes too large. This leads to a dilemma, in which frequent remote sensing will provide detailed data of growth processes of plants to the improvement of GP accuracy, but incorporation of this data enlarges the dimension of the covariance matrix of the multitrait GP model and hence decreases the accuracy due to the estimates of the parameters of the covariance matrix. In such cases, an appropriate dimension‐reduction method, which can be applied to longitudinal remote sensing data, may improve the accuracy of the multitrait GP model with a low dimensional covariance matrix.

**Core Ideas**

- Application of growth models and unmanned aerial vehicle (UAV) remote sensing in genomic prediction was investigated.
- The growth of soybean plants was estimated from canopy area and height using a UAV.
- Growth models were used to determine growth patterns of the breeding population.
- The inclusion of growth parameters improved prediction accuracy of biomass.

There are several statistical methods of dimension reduction, including a principal component analysis. However, there is no guarantee that the selected direction of data compression reflects the genetic divergence of growth curves. On the other hand, usually some empirical prior knowledge about the patterns of the growth curves can be obtained. The modeling of the variations in the patterns of growth curves based on prior knowledge may enable the effective extraction of growth characteristics as model parameters. Here we fitted simple growth models to remote sensing data and used model parameters as a representation of each growth curve. Several reports have shown that the use of growth models improved the results of QTL analyses (Ma et al., 2002; Wu et al., 2002). The use of growth parameters in the multitrait GP model is also expected to lead to an improvement in prediction accuracies.

Another possibility to improve the prediction accuracy of target traits is the use of a two‐step GP model. It should be noted that the two‐step GP model in this study does not indicate a two‐step model in which the genotypic values are estimated from the phenotype in the first step and predicted using the GP models in the second step. In the two‐step GP model, secondary traits were first predicted using GP, and target traits were identified using predicted values of secondary traits (Toda et al., 2020). Because this method estimates the direct linear relationship between secondary and target traits, instead of the covariance matrix between all traits, the two‐step GP model may be effective even when high‐dimensional remote sensing data is included.

Prediction accuracies of several models were compared in this study, in which soybean [*Glycine max* (L.) Merr.] biomass was chosen as the target trait. We estimated canopy height (CH) and area (CA) with UAV images, and simple growth models were fit to growth curves of the CH and CA to extract growth characteristics. The prediction accuracies of the biomass using GP, multitrait GP, and two‐step GP models were compared, using the observed values or growth parameters of CH and CA as secondary traits.

## 2 MATERIALS AND METHODS

### 2.1 Field trial

**FIGURE 1:**
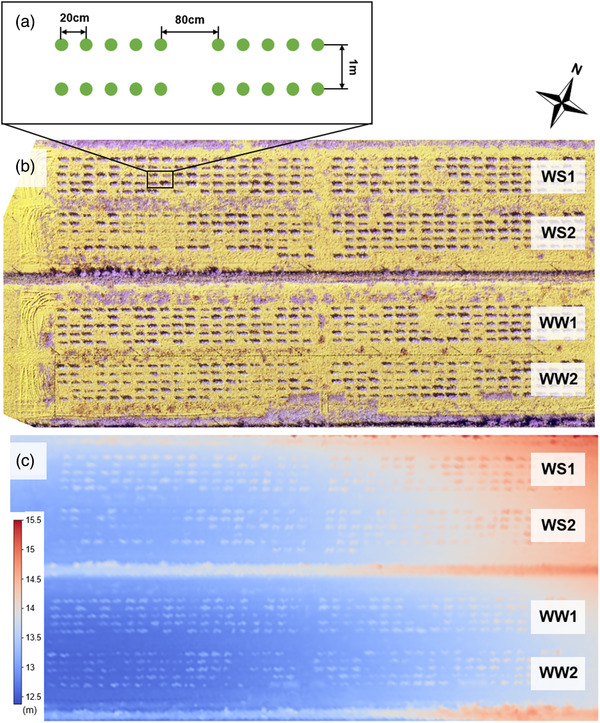
The layout of the plots of well‐watered (WW) and water‐stressed (WS) plants and the result of remote sensing. (a) The layout of plots. (b) An ortho‐mosaic image of the field, obtained on 24 Aug. 2016. The near‐infrared (NIR) information was used instead of blue color. (c) Digital surface model (DSM) of the field obtained on 24 Aug. 2016. The color represents the elevations of each point shown in a color bar

A total of 198 accessions of soybean core collections registered in the National Agriculture and Food Research Organization Genebank (https://www.gene.affrc.go.jp/index_en.php) were used in this study. In 2016, a field trial of the 198 accessions was conducted in an experimental field with sandy soil at the Arid Land Research Center, Tottori University (35°32′ N lat, 134°12′ E long, 14 m asl). Distances between each row, plot, and individual were 100, 80, and 20 cm, respectively (Figure 1a). Each plot consisted of five plants. Sowing was performed on 4 July 2016. Basal fertilizer (7.8, 9.6, 9.6, 11.2, and 40 g/m^2^ of N, P, K, Mg, and Ca, respectively) was applied to the field before sowing, and 25% of each value was applied as additional fertilizer on 30 Aug. 2016.

Plants were watered with sprinklers three times per day for 15 min each until 9 Aug. 2016. To evaluate the phenotypic variation between different environmental conditions, plants were divided into two treatments: well‐watered (WW) and water‐stressed (WS). On 10 Aug. 2016, plants in the WW treatment group were watered with sprinklers about once every 2 d, whereas plants in the WS treatment were watered about once every 4 d. The experimental design was similar to the split‐plot design; two replications with randomized plots were taken for each treatment, but the places of cultivars were the same between the corresponding replications of treatments (WW1/WS1, WW2/WS2) (Figure 1b–c).

### 2.2 Remote sensing and destructive measurements

Unmanned aerial vehicle remote sensing began 2 weeks after sowing and was performed about once every 10 d. A red‐green‐NIR (near‐infrared) camera (DJI Zenmuse X3 Camera Green‐Red‐NIR 800–900 nm, LDP) mounted on a consumer UAV (DJI Inspire 1) was used for the image collection. The UAV flew 12 m above the ground and took images at 2‐s intervals using an autofocus function. A single UAV flight took about 10 min and collected 500–600 images from each treatment region. The height of plants cultivated in WW1 was measured manually with rulers on the same day of remote sensing as the ground‐truth of the CH.

Destructive sampling was conducted from 13 to 15 Sept. 2016, to measure the biomass of plants (fresh weight of the above‐ground part of a plant) in the entire experiment to use as a target trait for prediction. Three plants were selected to measure the biomass in each plot. Also, images of leaves separated from the stems were taken with a camera (7R, SONY) with a 35‐mm lens (SEL35F28Z, SONY) to estimate the total leaf area of a plant and to compare with CA measured by UAV remote sensing. The camera had been filter removed to take a red‐green‐NIR image. Images of leaves were taken only for plants cultivated in WW1. The estimated leaf area was compared with the values of the CA obtained by remote sensing.

### 2.3 Estimation of traits using remote sensing data

Digital surface models (DSM) and ortho‐mosaic images of the field on each observation date were constructed (Figure 1b–c) using the images collected by UAV remote sensing and Pix4Dmapper software (Pix4D). Constructions of 3D data failed on some dates due to the difficulty in finding matching points of reference between several images. Individual plots were segmented out from the DSM and ortho‐mosaic images using geolocation information. Then, regions of the canopies and the ground were segmented out from the ortho‐mosaic images of an individual plot via the thresholding of the ortho‐mosaic images based on normalized difference vegetation index. Finally, the CH and CA of each plot were estimated based on the DSM and ortho‐mosaic images, respectively. The CH was estimated as the difference in mean values of ground elevation in each plot and the top elevation of the canopy in the DSM. The CA was estimated as the area of the canopy projected in the ortho‐mosaic images. Python 2.7 (https://www.python.org) was used for image analysis, and R 3.5.2 (R Core Team, 2020) was used for the other analyses below.

Canopy height measured with the image analysis had such a large bias that longitudinal growth patterns were hardly observable. We corrected CH using the manually measured plant height of a subset of plots. We assumed that CH measured by UAV was modeled as

(1) $$CH_{ij}\;=\;a_{j}PH_{ij}\;+\;e_{ij}$$

where CH*_ij_* is the CH measured by the UAV of the *i*th plot on the *j*th observation date, PH*_ij_* is plant height measured manually, *e_ij_* is the error of estimation of the *i*th plot on the *j*th observation date, and *a_j_* is a bias in the estimation of CH due to error inherent in the construction of the DSM. We estimated the bias *a_j_* based on the regression and then obtained CH calibrated by the bias (i.e., CH*_ij_*/*a_j_*). The measurement accuracy of the CH or calibrated CH was evaluated with root mean squared errors (RMSEs):

(2) $$\mathrm{RMSE}\;=\sqrt{\frac{1}{N}\sum_{i,j}{\left(y_{ij}-PH_{ij}\right)}^{2}}$$

where *y* is the CH or calibrated CH, and *N* is the number of data points. Because the measurement errors of CH were almost proportional to its values, another index was also used: RMSE divided by the mean of the calibrated CH (ratio of RMSE to mean values, RRMSE),

(3) $$\mathrm{RRMSE}\;=\mathrm{RMSE}/\bar{\mathrm{PH}}$$

where $\bar{\mathrm{PH}}$ is the mean plant height value. Although RMSE was affected by the mean plant height values, RRMSE was assumed to be a stable index. The calibrated CH was used as the phenotypic value in subsequent analyses. As plant height was not measured manually around 5 or 6 Sept. 2016, the correction of CH measured by UAV was not performed for data collected on those dates. Also, CH observed on 10 Aug. 2016 was removed in the following analysis due to lack of reliability (Supplemental Figure S1).

### 2.4 Growth model of longitudinal traits

**FIGURE 2:**
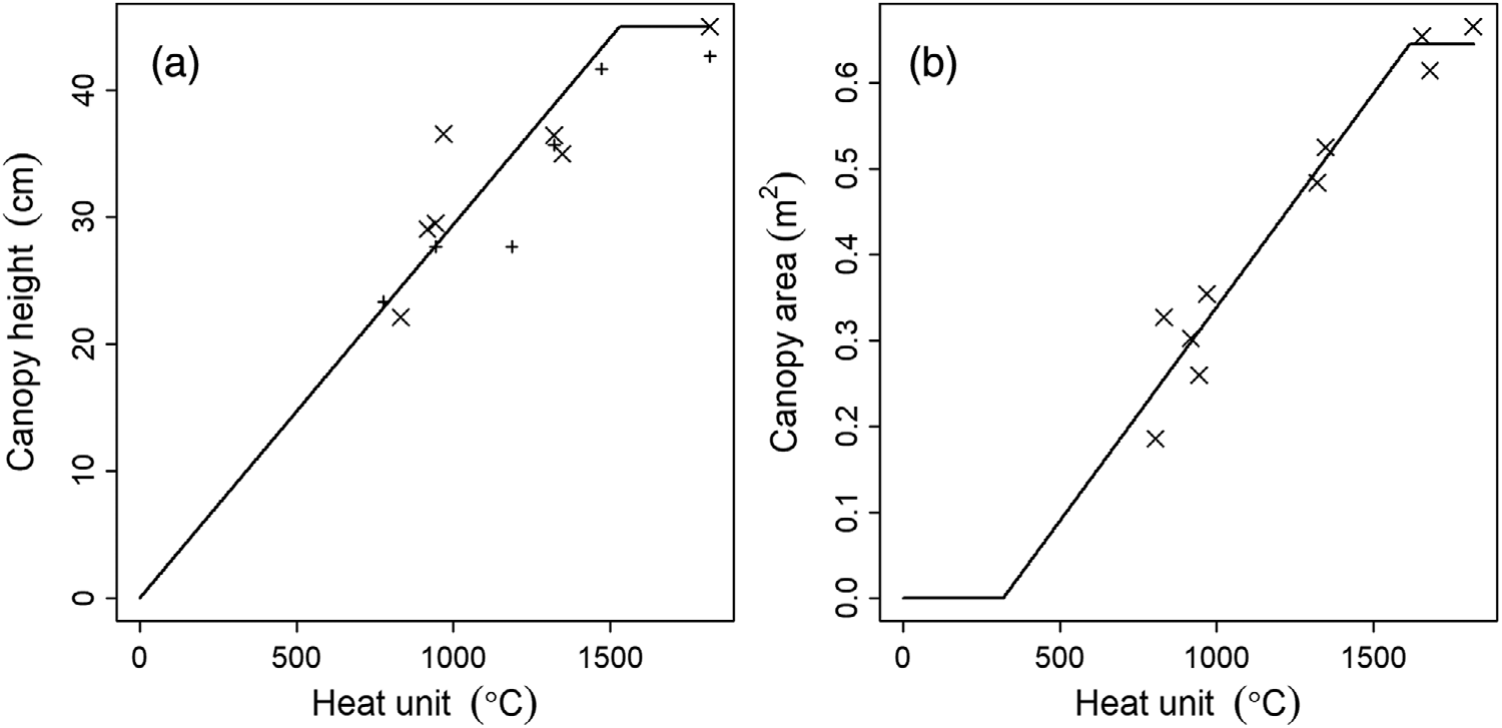
Examples of growth models of canopy height (a) and canopy area (b). The canopy height and area of a plot in WW2 are plotted as crosses (×), whereas the plant height with manual measurement is plotted as small plus marks (+). The solid lines represent the fitted segmented regression lines of the growth models. WW, well‐watered

To model growth patterns of CH and CA, we fitted segmented regression lines to the longitudinal data. The cumulative temperature after the sowing date, or heat unit (HU), was used as a dependent variable. The segmented regression model for the CH was

(4) $$\;y_{\mathrm{ij}}=\left\{\begin{array}{c} k_{i}{\mathrm{HU}}_{j}\;\;\text{if}\;{\mathrm{HU}}_{j}<\mathrm{GT}P_{i}\\ {\mathrm{ymax}}_{i}\;\;\text{if}\;{\mathrm{HU}}_{j}\geq \mathrm{GT}P_{i} \end{array}\right.$$

where *y_ij_* is the CH of the *i*th plot on the *j*th observation date; HU*_j_* is the HU (cumulation of daily mean temperatures [°C]) on the *j*th observation date; *k_i_* is the growth speed of the canopy height of the *i*th plot; GTP*_i_* is the value of the HU at the growth termination point (GTP) of the *i*th plot; ymax*_i_* is the maximum value of the CH of the *i*th plot. The segmented regression model for the CA is

(5) $$\;y_{\mathrm{ij}}=\left\{\begin{array}{c} 0\;\;\text{if}\;{\mathrm{HU}}_{j}<\mathrm{GS}P_{i}\\ k_{i}\left({\mathrm{HU}}_{j}-\mathrm{GS}P_{i}\right)\;\;\text{if}\;\mathrm{GS}P_{i}\leq {\mathrm{HU}}_{j}<\mathrm{GT}P_{i}\\ \mathrm{yma}x_{i}\;\;\text{if}\;{\mathrm{HU}}_{j}\geq \mathrm{GT}P_{i} \end{array}\right.$$

where *y_ij_* is the CA of the *i*th plot on the *j*th observation date, GSP*_i_* is the HU at the growth start point (GSP) of the *i*th plot, and the other variables are the same as those in Equation 4. Figure 2 illustrates the models fitted to the data of the WW treatment group. These growth parameters (*k_i_*, GTP*_i_*, and ymax*_i_* for CH and *k_i_*, GSP*_i_*, GTP*_i_*, and k*_i_* for CA) were estimated by fitting the growth models to UAV remote sensing measurement data with the least‐squares method.

### 2.5 Genotypic values of traits and parameters

Genotypic values of all traits (biomass, leaf area, CH, and CA) and growth parameters were estimated for use in GP. For each trait and treatment, we fitted the following mixed model:

(6) $$y_{ij}=\beta_{i}\;+u_{j}+e_{ij}$$

where *y_ij_* is the phenotype value of the *i*th replicate and *j*th genotype, β*_i_* is the effect representing the differences between the replicates, *u_j_* is the random genotypic value, and *e_ij_* is the residual value. In the vector form,

(7) y=Xβ+Zu+e

where **y** is a vector of the phenotype, **β** is a vector of replicate effects, **u** is a vector of genotypic values (with the assumption that was yielded from the Gaussian probability distribution **u** ∼ N(0, σ_u_^2^ **I**), where σ_u_^2^ is a genetic variance and **I** is an identity matrix), **e** is a vector of residuals (with the assumption **e** ∼ N(0, σ_e_^2^ **I**) where σ_e_^2^ is a residual variance), and **X** and **Z** are design matrices. The R package lme4 (version 1.1‐20) was used to solve for Equation 7 and best linear unbiased prediction of the genotypic values (**u**) were obtained. For CH and CA, Equation 7 was applied separately for each observation date.

### 2.6 Genomic relationship matrix and heritability

Whole‐genome sequencing data of all 198 accessions were available and used to estimate the genomic relationship matrix. The details about the DNA extraction and whole‐genome sequencing techniques were provided by Kanegae et al. (2021). Only the biallelic sites in all accessions with a minor allele frequency ≥0.025 and missing rate <0.05 were extracted, and the missing genotypes were imputed. This genotyping data identified 4,776,813 single nucleotide polymorphisms. Genotypes for individual alleles were represented as −1 (homozygous for the reference allele), 1 (homozygous for the alternative allele), or 0 (heterozygous for the reference and alternative alleles). A genomic relationship matrix **A** was estimated as **A** = **XX** ^T^/*c*, where **X** is an *n* × *m* marker genotype matrix (*n* and *m* are the numbers of lines and markers, respectively), and *c* is the normalization constant (Endelman & Jannink, 2012). Then, the narrow‐sense heritability was estimated for all traits with the genomic best linear unbiased prediction model,

(8) y=μ+Zg+e

where **y** is a vector of genotypic values (best linear unbiased predictions from Equation 7), **μ** is a vector of mean values, **g** is a vector of random genetic effect (with the assumption **g ∼** N[0, σ_g_^2^ **A**]), **e** is a vector of residuals (with the assumption **e ∼** N[0, σ_e_^2^ **I**]), and **Z** is a design matrix. The R package rrBLUP (version 4.6) (Endelman, 2011) was used to solve for Equation 8. After solving this mixed model, narrow‐sense heritability was estimated as *h* ^2^ = σ_g_^2^/(σ_g_^2^ + σ_e_^2^).

### 2.7 Genomic models predicting biomass

**FIGURE 3:**
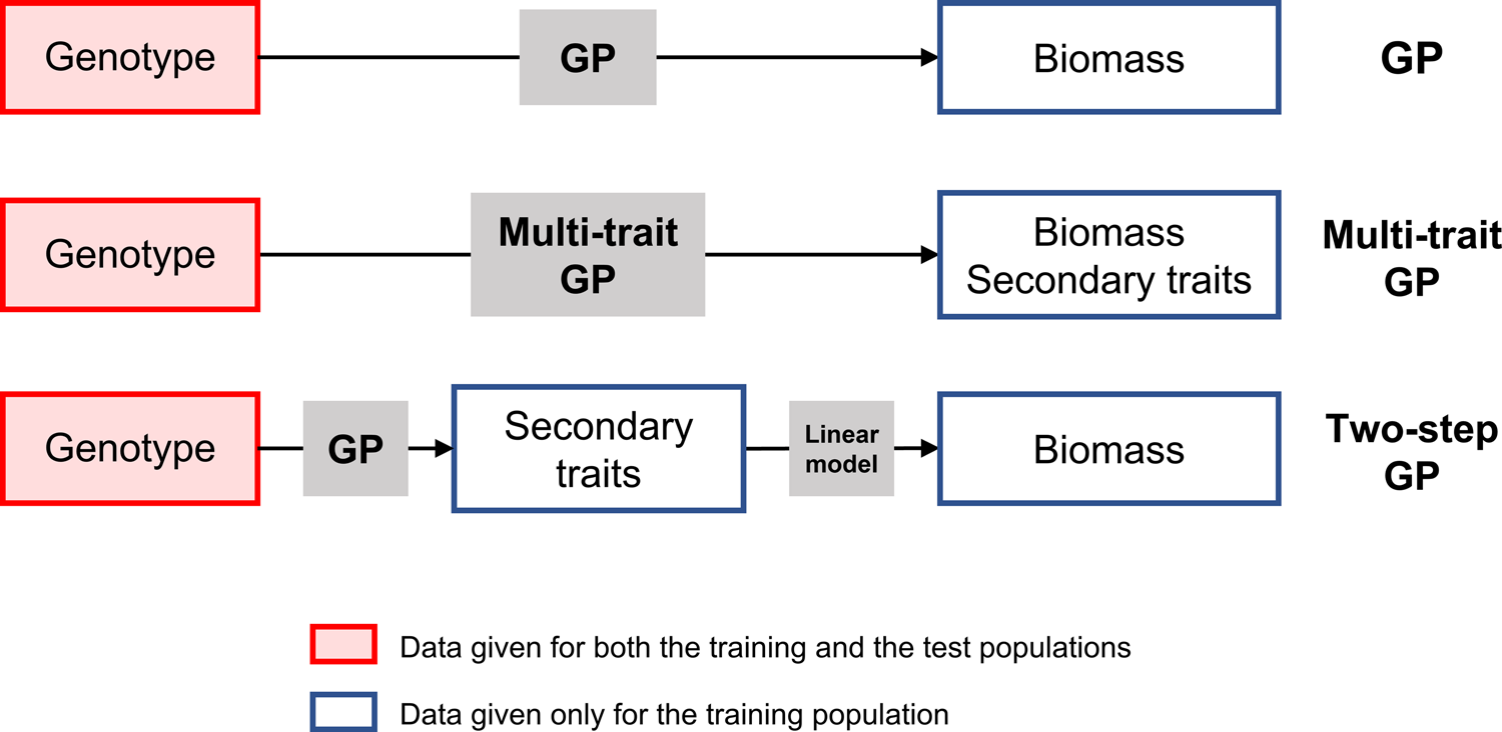
Comparison of the structures of three prediction models: genomic prediction (GP), multitrait GP, and two‐step GP models. The relationship between genotype data, secondary traits (the traits measured by unmanned aerial vehicle remote sensing or growth parameters), and biomass are shown

Three types of models were constructed to predict biomass (Figure 3): GP, multitrait GP, and two‐step GP models. The GP model was the same as the model shown in Equation 8, where only the data of the genotypic values of biomass was used in the prediction.

The multitrait GP model was an extension of the GP model and attempted to calculate variances and covariances of several traits simultaneously (Calus & Veerkamp, 2011; Jia & Jannink, 2012). If secondary traits, which are involved in a model along with biomass, are both highly correlated with biomass and demonstrate high heritability, incorporation of these traits is expected to improve the prediction accuracy of biomass (Calus & Veerkamp, 2011). This model can be expressed as

(9) $$\left(\begin{array}{c} y_{1}\\ \vdots \\ y_{D} \end{array}\right)=\left(\begin{array}{c} \mu_{1}\\ \vdots \\ \mu_{D} \end{array}\right)\;+\;\left(\begin{array}{ccc} Z_{1} & \;\cdots & \;0\\ \vdots & \;\ddots & \;\vdots \\ 0 & \;\cdots & \;Z_{D} \end{array}\right)\left(\begin{array}{c} g_{1}\\ \vdots \\ g_{D} \end{array}\right)+\left(\begin{array}{c} e_{1}\\ \vdots \\ e_{D} \end{array}\right)$$

where *D* is the number of traits in the model, and **y** *_d_*, **μ** *_d_*, **g** *_d_*, and **e** *_d_* are vectors of genotypic values, mean values, random genetic effects, and residuals of *d*th trait, respectively. Assumptions for random effects were included in the model: (**g**_1_^T^, …, **g** *_D_* ^T^)^T^ ∼ N[**0**, **A ⊗ H**] and (**e**_1_^T^, …, **e** *_D_* ^T^)^T^ ∼ N[**0**, **I** ⊗ **R**], where **H** is a genomic variance‐covariance matrix between traits, **R** is a residual variance‐covariance matrix between traits, and ⊗ is the Kronecker product of matrices. The R package EMMREML was used to solve Equation 9, in which the high‐speed approximation algorithm EMMA (Kang et al., 2008) was used. We tested two models, one in which the measured values of CH and CA were used as secondary traits. The number of traits in model *D* was equal to the sum of the number of observation dates for CH and CA, which were 16 and 13 in the WW and WS treatments, respectively. The other in which the growth parameters estimated by model (Equations 4 and 5) were used. In this model, *D* was equal to the number of growth parameters for CH and CA (*D *= 7). The observed values of secondary traits in the training population were used to build a prediction model, while the observed values of secondary traits in the test population were not used in the prediction of biomass in the test population.

The two‐step GP model used a two‐step approach. It should be noted that the two‐step GP model in this study does not indicate a two‐step model in which the genotypic values are estimated from the phenotype in the first step and predicted using the GP models in the second step. In the first step of the two‐step GP model in this study, secondary traits were predicted using univariate GP. In the second step, biomass was calculated from predicted secondary traits using multiple regression analysis. Genetic values of biomass and those of secondary traits in the training population were used to estimate coefficients of multiple regression, whereas the values of secondary traits in the test population predicted by GP were used to predict values of biomass in the test population. Note that only measured values of secondary traits of the training sets were used; phenotypic values of secondary traits were not used in the prediction for the test set by replacing them with genomic predicted values. To compare with the multitrait GP model, we tested two cases, one in which the observed values of CH and CA were used as the secondary traits, and one in which the growth parameters estimated by model (Equations 4 and 5) were used.

Ten‐fold cross‐validation with random data splitting was repeated 10 times to validate the prediction accuracies of the prediction models. Prediction accuracies were evaluated using correlation coefficients of the genotypic values from Equation 7 and predicted values.

## 3 RESULTS

### 3.1 Estimation accuracy of crop traits by UAV remote sensing

**FIGURE 4:**
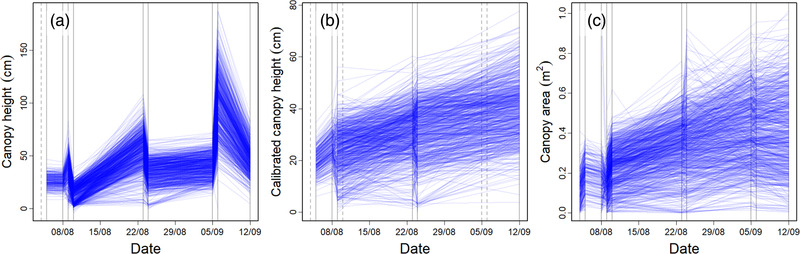
Longitudinal patterns of the canopy height and canopy area. (a) Canopy height obtained from the image processing, (b) canopy height corrected with plant height manually measured, and (c) canopy area. Vertical lines indicate the date of unmanned aerial vehicle remote sensing; solid lines indicate the dates when data were available, whereas dashed lines indicate the dates when data were unavailable due to failure in the construction of digital surface models in Pix4Dmapper

The estimation bias in CH measured by the image processing was so large that the longitudinal tendency of growth could not be tracked (Figure 4a). However, the measured values of CH were correlated with the values of plant height measured manually on each observation date (Supplemental Figure S1). Upon correction of the CH using the manually‐measured plant height and model (1), the estimation bias decreased, as shown by RMSE and RRMSE values (Table 1). The RRMSE of the calibrated CH on 10 Aug. 2016 is exceptionally higher than the other results due to the failure in DSM estimation (Supplemental Figure S1). After the calibration and removal of the CH data on 10 Aug., the growth processes were tracked (Figure 4b).

**TABLE 1:** Estimation accuracy of canopy height

			**CH**		**Calibrated CH**	
**Date of UAV remote sensing**	**Date of manual measurement**	**Number of samples**	**RMSE**	**RRMSE**	**RMSE**	**RRMSE**
5 Aug.	3 Aug.	87	7.17	0.379	2.85	0.151
8 Aug.	9 Aug.	88	4.13	0.167	4.00	0.161
9 Aug.	9 Aug.	176	12.45	0.519	5.43	0.227
10 Aug.	9 Aug.	178	12.87	0.537	10.86	0.455
23 Aug.	23 Aug.	177	26.95	0.822	7.45	0.228
24 Aug.	23 Aug.	177	7.09	0.216	6.85	0.210
12 Sept.	12 Sept.	174	9.35	0.239	7.43	0.190

*Note*. The values of canopy height (CH) obtained from image processing and CH calibrated with the plant height were used to evaluate estimation accuracy. The number of plant height samples available for calibration, root mean squared errors (RMSE), and RMSE divided by the mean of CH (RRMSE) are shown. The nearest dates of manual measurement from the dates of unmanned aerial vehicle (UAV) remote sensing were chosen for comparison.

**FIGURE 5:**
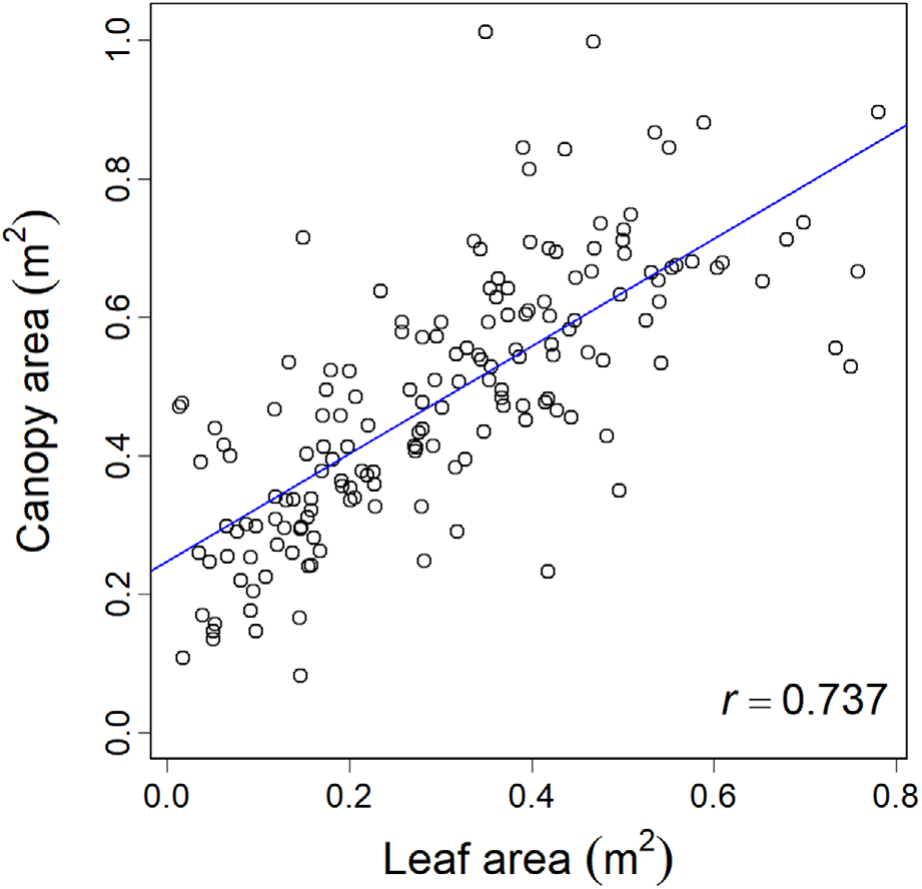
Comparison of canopy area measured using unmanned aerial vehicle remote sensing and leaf area measured with destructive sampling. The values of the leaf area of one plant are plotted on the horizontal axis, whereas the values of the canopy area of one plot are plotted on the vertical axis. The blue line represents a regression line of canopy area to leaf area. The correlation coefficient (*r*) of the leaf and canopy areas is shown in the plot

Compared with the CH, the growth pattern in the CA was clear without correction (Figure 4c). The CA as measured by UAV remote sensing at harvest and the leaf area as measured in the destructive sampling demonstrated a significant correlation (adjusted coefficient of determination *R* ^2^ = 0.540, *p* < 1.0 × 10^−10^) (Figure 5).

### 3.2 Correlation with biomass and heritability of traits as measured by UAV remote sensing

**FIGURE 6:**
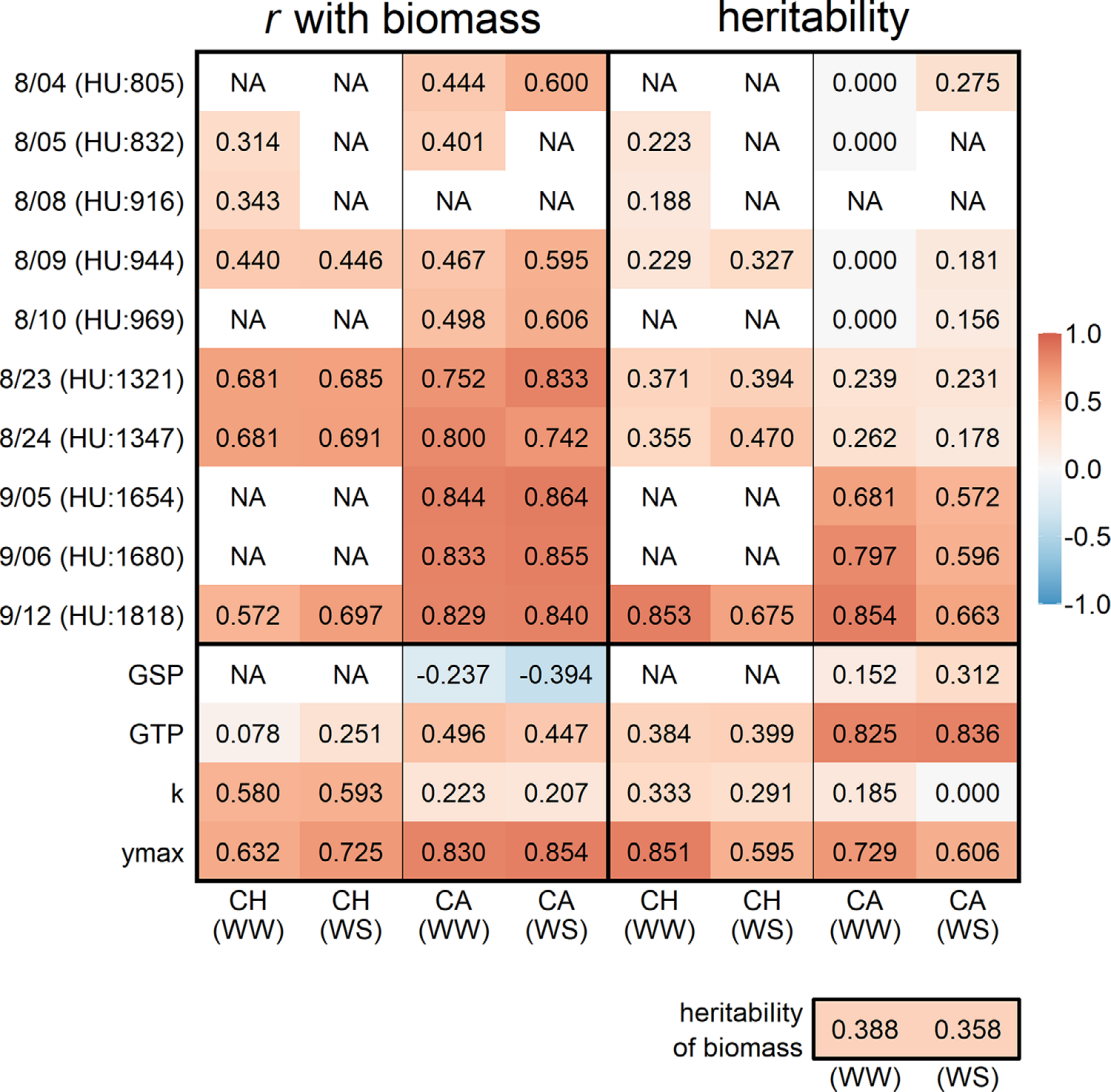
A heatmap of correlation coefficients between canopy height/area (CH/CA) and biomass, and narrow‐sense heritability of CH/CA and biomass. The values of well‐watered and water‐stressed treatment groups (WW/WS) are shown separately. The heat units (HUs) of each date are written in parentheses. GSP, growth start point; GTP, growth termination point; ymax, maximum value of the CH

Correlation coefficients between CH and biomass ranged from 0.314 to 0.697, and those between CA and biomass ranged from 0.401 to 0.864 (Figure 6). Those values tended to increase closer to the date of destructive sampling and to display higher values in CA than in CH.

Canopy height and CA showed increasing heritability over time, ranging from 0.188 to 0.853 in CH and from 0.000 to 0.854 in the CA. The CA of the WW treatment group in the early growth stage showed notably low heritability due to effect of competition with weeds. On the other hand, the heritability of the CH and CA was higher in the WW treatment than in the WS treatment in the later growth stage.

### 3.3 Genetic or biological meanings of growth model parameters

The correlation between the phenotypic values measured by UAV remote sensing and the parameters of growth models showed various patterns in growth parameters (Supplemental Figure S2). The correlation between ymax and the observed values of CH and CA increased over time, as did GTP. The correlation between *k* and the observed values was stable over the observed time period.

The correlation coefficient between ymax and biomass was the highest in the parameters of CH and CA (Figure 6). The heritability of the ymax of the CH and GTP of the CA was the highest among all parameters, whereas *k* of CH and GSP or *k* of the CA was the lowest.

**FIGURE 7:**
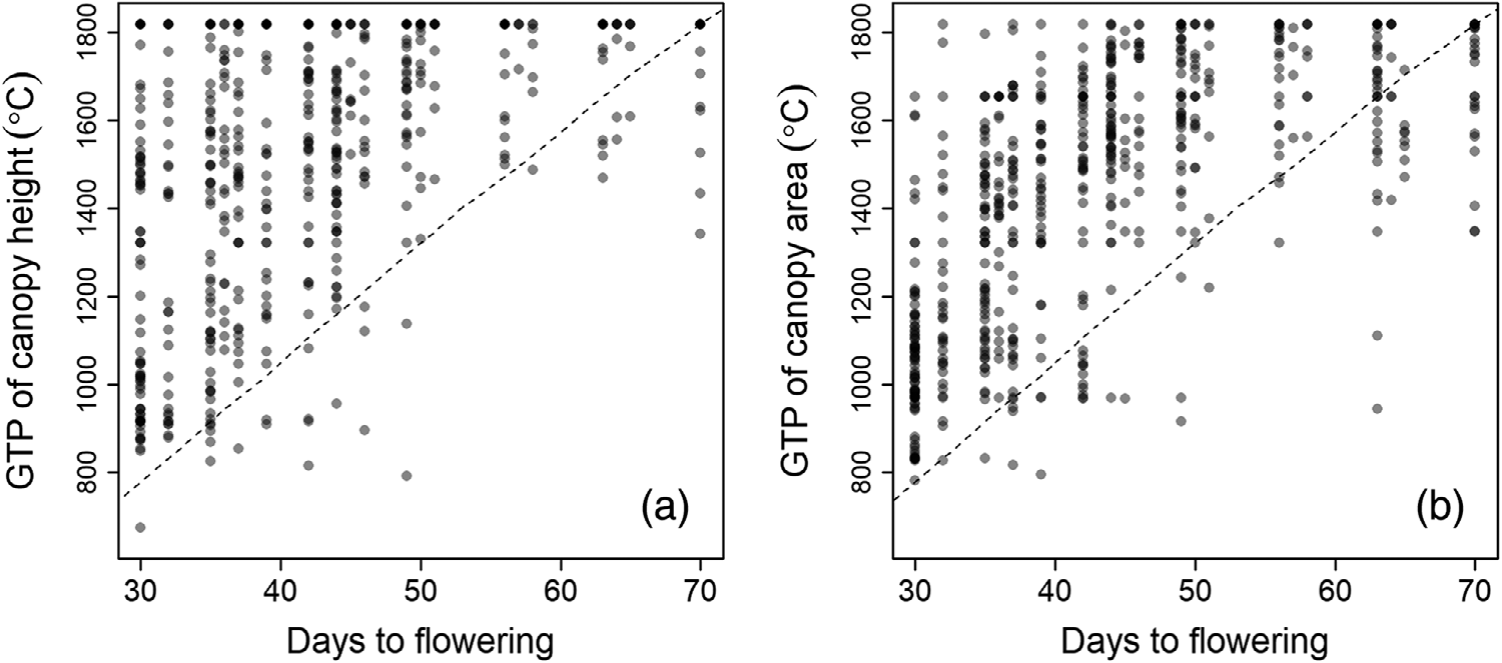
Comparison of growth termination point (GTP) of the canopy height (a) and area (b) and days to flowering. Dotted lines indicate the correspondence of the date and heat unit

By comparison with the flowering date, it was found that GTP of both CH and CA tended to be longer than the number of days to flowering; in other words, termination of growth of CH and CA were estimated to be later than their flowering (Figure 7).

### 3.4 GP

**FIGURE 8:**
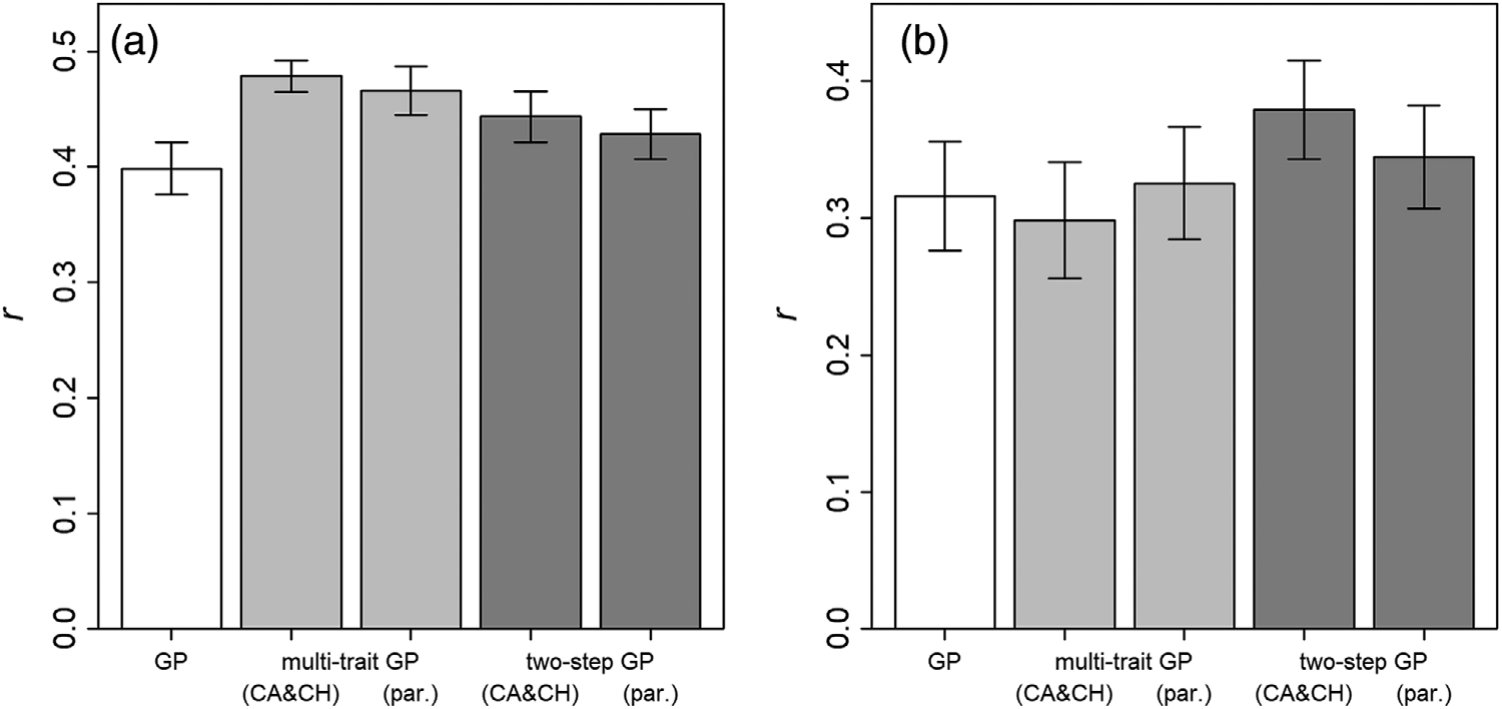
Prediction accuracies of biomass of genomic prediction (GP); multitrait genomic prediction with canopy height (CH) and area (CA) or growth parameters (par.) as the secondary traits; and two‐step genomic prediction with CH, CA, and par. as the secondary traits. Correlation coefficients of observed and predicted values were plotted. The accuracies of well‐watered and water‐stressed treatment groups are shown in (a) and (b), respectively. Error bars indicate SD

In the WW treatment group, the prediction accuracy of the multitrait GP model using the CH and CA as the secondary traits was the highest (*r* = 0.478). On the other hand, in the WS treatment group, the prediction accuracy of the two‐step GP model using the CH and CA was the highest (*r* = 0.379, Figure 8). Accuracies of the models using the growth parameters were lower than the models using the observed data of the CH and CA, with exception of the accuracy of the multitrait GP model in the WS treatment group, for which the accuracy of the multitrait GP model using the CH and CA was notably low.

The regression coefficients of the second step of the two‐step GP model are visualized in Supplemental Figure S3. The coefficients of the measured values of the CA just before harvest are high. The coefficients of the CH in the early growth stage are only high in the WW treatment.

## 4 DISCUSSION

### 4.1 Acquisition of longitudinal growth traits through UAV remote sensing

A large estimation bias was observed in CH due to the effects of wind or change in radiation levels. Such bias, which is inevitable in the field of remote sensing, hinders and prevents the tracking of detailed plant growth process. Georeferenced ground control points, which were not installed in our study, might help more precise evaluation of height information (Singh et al., 2019). However, it was shown that the calibration using manual measurements can greatly reduce this bias. Hu et al. (2018) also showed that the manual calibration increased the measurement accuracy of the height of sorghum [*Sorghum bicolor *(L.) Moench], even when the ground control points were installed in the field. As plant height is strongly related to biomass (Bendig et al., 2015), such a calibration method can play an essential role in breeding to observe the longitudinal growth of plants.

On the other hand, the CA showed lower bias than CH and did not require any correction with manual measurements, suggesting that CA can be a robust measurement of temporal growth of soybean plants. Also, the correlation between the CA and biomass was high from an early stage of growth (Figure 6). A similar trait, projected leaf area, was already shown to be highly correlated with biomass in a high‐throughput phenotyping platform (Golzarian et al., 2011). In previous studies on field remote sensing, the mean values of the vegetation indices of images were calculated for each plot and used in the analysis (Duan et al., 2017; Rutkoski et al., 2016; Tattaris et al., 2016). However, trait estimation based on the segmentation of plants, such as CA, is also a suitable trait to observe plant conditions due to their robustness and correlation with biomass.

### 4.2 Growth model

Large differences were observed among the heritability of the growth parameters of the CA (Figure 6). The parameters *k* and GSP demonstrated low heritability, possibly because these parameters reflected the influence of the competition with weeds during the early growth period. On the other hand, high heritability was observed in the parameters GTP and ymax, indicating that these parameters reflected the genetic characteristics of growth patterns of the CA. ymax was especially suitable as a secondary trait in GP, because of its high heritability and correlation with biomass. The growth parameter CH showed varied heritability, whereas ymax had high heritability and correlation with biomass but no parameter showed higher heritability and correlation with biomass than those of GTP of CA.

Focusing on the difference between the two treatments, the heritability of the CH and CA is lower in the WS treatment than in the WW treatment (Figure 6), possibly due to drought stress, which increases the magnitude of environmental effects on the plant phenotype. This tendency is also reflected in the heritability of the growth parameters, which indicates that the growth models could reflect the environmental and genetic variations in growth patterns.

It was also shown that the GTP of CH and CA was later than flowering (Figure 7). This result matches a characteristic of soybean, of which the stems and leaves keep growing after the beginning of flowering. The growth models of Equations 4 and 5 were found to appropriately extract these growth characteristics from UAV remote sensing data.

### 4.3 Prediction accuracies of multitrait and two‐step GP models

Both multitrait and two‐step GP models could improve the prediction accuracy of GP (Figure 8). This improvement was not observed in previous studies in cases where secondary traits were unavailable in the test population (Rutkoski et al., 2016; Sun et al., 2017). One reason that these models improved accuracy in this study is the high heritability of the secondary traits, especially the CA. It was reported that the multitrait GP model could improve the prediction accuracy if secondary traits were highly correlated with the target trait and also display high heritability (Calus & Veerkamp, 2011); CA was found to be a good secondary trait in predicting biomass from this point of view (Figure 6).

Comparing the results of the multitrait and two‐step GP models, the prediction accuracies were higher in the multitrait GP model in the WW treatment. This is because multitrait GP estimates the random genetic effect of multiple traits simultaneously, whereas two‐step GP does so for each trait separately. However, the accuracy of the multitrait GP model using the CH and CA was particularly low in the WS treatment group, and the accuracy of the two‐step GP model using the CH and CA exceeded it. This was due to the failure of estimation of covariance structures (underestimation of genetic variances, Supplemental Figure S2) in the multitrait GP model, which was suggested by the lower heritability of biomass in the multitrait GP model using the CH and CA than in the genomic best linear unbiased prediction of Equation 7 (Supplemental Figure S4). An increase in the number of secondary traits can also cause such estimation failures. On the other hand, the prediction accuracy of the two‐step GP model was assumed to be stable, because it does not require complex computation. Two‐step GP is a robust approach to incorporate an increased number of secondary traits.

The regression coefficients of the second step in the two‐step GP model indicated that the CA measured just before harvest or growth parameter ymax of CA had much importance in the prediction of biomass (Supplemental Figure S3). This result can be expected since the CA at the harvest time is strongly correlated with biomass (Figure 6). It is also shown that the CH in the early growth stage is useful in predicting the biomass in the WW treatment. One interpretation of this result is that the CH in the early growth stage can reflect its growth rate (*k*), which is included in the two‐step GP model (Supplemental Figure S3, right panels). Another interpretation is multicollinearity, which may be avoided with the application of a more advanced regression model such as partial least‐squares regression.

Linear regression was used in the second step of the two‐step GP model in this study, but other statistical approaches can alternatively be used (Toda et al., 2020). If the correlation among the secondary traits is high, partial least‐squares regression or principal component regression are recommended for the second step of two‐step GP to avoid a problem with multicollinearity. Also, nonlinear models, such as random forest (Breiman, 2001), or support vector machines can be used to include a nonlinear relationship between the secondary traits and the target traits in the final model.

### 4.4 Use of growth parameters in the prediction model

The prediction accuracies of the multitrait and two‐step GP models using the CH and CA as the secondary traits were generally higher than those of the multitrait and two‐step GP models using the growth parameters. The reduction in prediction accuracies when using the growth parameters likely occurred because the information in the original remote sensing data was partially lost when growth models were fitted. However, the prediction accuracy of the multitrait GP model using the growth parameters in the WS treatment group was higher than that of the multitrait GP model using the CH and CA (Figure 8), although the heritability of the biomass estimated with the model was low (Supplemental Figure S4). Both multitrait GP models could not estimate the covariance matrix in this case, but the high heritability of the growth parameters (Figure 6) increased the prediction accuracy in the multitrait GP model using the growth parameters. Although the prediction accuracy of the multitrait GP model using the growth parameters was not different from that of GP, it indicated that the incorporation of a growth model could effectively compress the longitudinal traits as growth parameters and improve the predictive ability of the multitrait GP model.

One alternative approach to incorporate longitudinal remote sensing data is a random regression model, which is a statistical method to consider the longitudinal structure of traits in GP. The time‐dependent structure of a covariance matrix is included in the model by explaining growth curves with base expansions using arbitrary functions (Kirkpatrick & Heckman, 1989). Several reports have shown that random regression was an effective method when applied to longitudinal remote sensing data (Anche et al., 2020; Sun et al., 2017) or growth data measured by a high‐throughput phenotyping platform (Campbell et al., 2018). Random regression can be understood as a dimension reduction of growth curves using functions such as Legendre polynomials or spline functions. Such functions have little restriction on their forms and are applicable to a wide range of growth patterns. Still, even in exchange for flexibility, it is challenging to include prior information about the target growth pattern in random regression. One advantage of our approach using growth models is that we could include prior knowledge about growth directly in the model, which led to the effective extraction of characteristics of the growth curves. Validation of the prediction ability of these methods using various datasets can help in choosing a more appropriate method when applied to real datasets.

We used simple growth models to express the growth of the CH and CA, but more complex models such as crop growth models can also be included in our framework. Crop growth models are simulation models where crop yield is expressed as an accumulation of carbon production during the cultivation period. Some reports have applied crop growth models in the framework of GP (Technow et al., 2015; Toda et al., 2020). Also, several methods have been developed to combine crop growth models and longitudinal remote sensing data via data assimilation (Jin et al., 2018; Kasampalis et al., 2018). It is expected that detailed growth characteristics can be estimated from longitudinal remote sensing data using sophisticated models such as crop growth models.

## 5 CONCLUSION

The modeling methods were investigated to analyze the soybean growth data measured using UAV remote sensing in plant breeding. In this study, we focused on the growth models of parameters that represent the growth characteristics of each plot or variety. Based on the statistical analysis of whole‐genome marker data, parameters of growth models helped improve the accuracy of multitrait or two‐step GP. Considering the future of plant breeding, it is expected that the development and spread of sensing technologies will enrich the plant phenotype data targeting their growth. However, applying conventional models in large phenotype data sometimes fails; for example, a decrease in the accuracy of multitrait GP in which CH and CA were incorporated (Figure 8). As the growth model and two‐step GP were developed in this study, a continuous development of analytical methods as well as effort to collect phenotype data is needed to increase selection efficiency and accelerate plant breeding.

## AUTHOR CONTRIBUTIONS

Yusuke Toda: Conceptualization; Data curation; Formal analysis; Investigation; Methodology; Software; Validation; Visualization; Writing‐original draft. Akito Kaga: Investigation; Resources; Writing‐review & editing. Hiromi Kajiya‐Kanegae: Data curation; Resources; Writing‐review & editing. Tomohiro Hattori: Investigation; Writing‐review & editing. Shuhei Yamaoka: Investigation; Writing‐review & editing. Masanori Okamoto: Investigation; Resources; Writing‐review & editing. Hisashi Tsujimoto: Funding acquisition; Resources; Writing‐review & editing. Hiroyoshi Iwata: Conceptualization; Funding acquisition; Investigation; Project administration; Supervision; Writing‐review & editing.

## CONFLICT OF INTEREST

The authors declare that the research was conducted in the absence of any commercial or financial relationships that could be construed as a potential conflict of interest.

## Supporting information


**Figure S1**. Comparison of the canopy height, as measured by UAV remote sensing, and plant height, measured manually. Non‐calibrated and calibrated canopy heights are plotted in the left and right panels, respectively. The number of data points (n), RMSE (root mean square error), and RRMSE (the ratio of RMSE to the mean) are indicated in the figure.

**Figure S2**. Longitudinal pattern of correlation coefficients between observed UAV remote sensing values and growth parameters. GSP, GTP, k, and ymax correspond to the growth starting point, growth termination point, growth speed, and maximum value, respectively. The results of canopy height and canopy area are shown. Solid and dashed lines indicate the results of WW and WS treatments, respectively. Vertical dashed lines indicate the date UAV remote sensing values were obtained.

**Figure S3**. Regression coefficients calculated in the second step of two‐step GP. The means and standard deviations of 100 coefficients (10‐fold cross‐validation and 10 repetition) are expressed as bars and arrows, respectively. The secondary traits are scaled in the second step, so the regression coefficients could be directly compared with each other. The results of two‐step GP using the canopy height (CH) and area (CA) or growth parameters (par.) in WW and WS treatments are shown in different panels.

**Figure S4**. Comparison of the prediction accuracy of biomass and its estimated heritability in each prediction. The square root of the heritability is used as the x axis, and the correlation coefficients of the observed and predicted values are used as the y axis. GP and multitrait GP models are used as the prediction models since the heritability of two‐step GP models cannot be estimated. The blue and red points indicate the results of the WW and WS treatments, respectively.
